# The Trichoptera barcode initiative: a strategy for generating a species-level Tree of Life

**DOI:** 10.1098/rstb.2016.0025

**Published:** 2016-09-05

**Authors:** Xin Zhou, Paul B. Frandsen, Ralph W. Holzenthal, Clare R. Beet, Kristi R. Bennett, Roger J. Blahnik, Núria Bonada, David Cartwright, Suvdtsetseg Chuluunbat, Graeme V. Cocks, Gemma E. Collins, Jeremy deWaard, John Dean, Oliver S. Flint, Axel Hausmann, Lars Hendrich, Monika Hess, Ian D. Hogg, Boris C. Kondratieff, Hans Malicky, Megan A. Milton, Jérôme Morinière, John C. Morse, François Ngera Mwangi, Steffen U. Pauls, María Razo Gonzalez, Aki Rinne, Jason L. Robinson, Juha Salokannel, Michael Shackleton, Brian Smith, Alexandros Stamatakis, Ros StClair, Jessica A. Thomas, Carmen Zamora-Muñoz, Tanja Ziesmann, Karl M. Kjer

**Affiliations:** 1 Beijing Advanced Innovation Center for Food Nutrition and Human Health, China Agricultural University, Beijing 100193, People's Republic of China; 2 College of Food Science and Nutritional Engineering, China Agricultural University, Beijing 100083, People's Republic of China; 3 Office of Research Information Services, Office of the Chief Information Officer, Smithsonian Institution, PO Box 37012, Washington, DC 20013-7012, USA; 4 Department of Entomology, University of Minnesota, 1980 Folwell Avenue, St Paul, MN 55108, USA; 5 School of Science, University of Waikato, Private Bag 3105, Hamilton 3240, New Zealand; 6 Grup de Recerca Freshwater Ecology and Management (FEM), Departament d'Ecologia, Facultat de Biologia, Institut de Recerca de la Biodiversitat (IRBio), Universitat de Barcelona, Diagonal, 643, 08028 Barcelona, Catalonia, Spain; 7 13 Brolga Crescent, Wandana Heights, Victoria 3216, Australia; 8 Department of Biology, Mongolian National University of Education, 3rd Palace, Beijing Street, Ulaanbaatar 14191, Mongolia; 9 44 Marks Street, Hermit Park, Queensland 4812, Australia; 10 Centre for Biodiversity Genomics, Biodiversity Institute of Ontario, University of Guelph, Guelph, Ontario, Canada N1G 2W1; 11 Environment Protection Authority Victoria, Ernest Jones Drive, Macleod 3085, Australia; 12 Department of Entomology, National Museum of Natural History, Smithsonian Institution, Washington, DC 20013-7012, USA; 13 SNSB-Bavarian State Collection of Zoology, Münchhausenstr. 21, 81247 Munich, Germany; 14 Büro H2-Ökologische Gutachten, Hess+Heckes GbR, Rumfordstraße 42, 80469 München, Germany; 15 Department of Bioagricultural Sciences and Pest Management, Colorado State University, 1177 Campus Delivery, Fort Collins, CO 80523, USA; 16 Biologische Station Lunz, Austrian Academy of Sciences, A-3293 Lunz am see, Austria; 17 Department of Plant and Environmental Sciences, Clemson University, PO Box 340310, Clemson, SC 29634-0310, USA; 18 Centre de Recherche en Sciences Naturelles Lwiro, P.O D.S Bukavu, D. R. Congo; 19 Senckenberg Biodiversity and Climate Research Centre, Senckenberganlage 25, 60325 Frankfurt am Main, Germany; 20 Unidad Multidisciplinaria de Docencia e Investigación, Universidad Nacional Autónoma de Mexcio, Facultad de Ciencias, Campus Juriquilla, Querétaro, 76230, México; 21 Finnish Environment Institute, Merikasarminkatu 8 D, 00160 Helsinki, Finland; 22 Illinois Natural History Survey, Prairie Research Institute at the University of Illinois at Urbana-Champaign, 1816 S. Oak Street, MC 652, Champaign, IL 61820, USA; 23 Aquatic Insects Expert Group of Finland, Siikinkatu 13, 33710, Tampere, Finland; 24 Murray-Darling Freshwater Research Centre, La Trobe University, 133 McKoy Street, Wodonga, Victoria 3691, Australia; 25 National Institute of Water and Atmospheric Research, PO Box, 11115, Hamilton 3240, New Zealand; 26 Scientific Computing Group, Heidelberg Institute for Theoretical Studies (HITS), 69118 Heidelberg, Germany; 27 Institute for Theoretical Informatics, Karlsruhe Institute of Technology, Karlsruhe, 35 D-69118 Heidelberg, Germany; 28 BioArch, Environment Building, Department of Biology, University of York, York, YO10 5DD, UK; 29 Department of Zoology, Faculty of Sciences, University of Granada, C/Severo Ochoa s/n, 18071 Granada, Spain; 30 Zoologisches Forschungsmuseum Alexander Koenig (ZFMK)/Zentrum fu¨r Molekulare Biodiversitätsforschung (ZMB), Bonn 5 76131 Karlsruhe, Germany; 31 Department of Entomology and Nematology, University of California-Davis, 1282 Academic Surge, Davis, CA 95616, USA

**Keywords:** DNA barcodes, caddisfly, phylogeny, integrative taxonomy

## Abstract

DNA barcoding was intended as a means to provide species-level identifications through associating DNA sequences from unknown specimens to those from curated reference specimens. Although barcodes were not designed for phylogenetics, they can be beneficial to the completion of the Tree of Life. The barcode database for Trichoptera is relatively comprehensive, with data from every family, approximately two-thirds of the genera, and one-third of the described species. Most Trichoptera, as with most of life's species, have never been subjected to any formal phylogenetic analysis. Here, we present a phylogeny with over 16 000 unique haplotypes as a working hypothesis that can be updated as our estimates improve. We suggest a strategy of implementing constrained tree searches, which allow larger datasets to dictate the backbone phylogeny, while the barcode data fill out the tips of the tree. We also discuss how this phylogeny could be used to focus taxonomic attention on ambiguous species boundaries and hidden biodiversity. We suggest that systematists continue to differentiate between ‘Barcode Index Numbers’ (BINs) and ‘species’ that have been formally described. Each has utility, but they are not synonyms. We highlight examples of integrative taxonomy, using both barcodes and morphology for species description.

This article is part of the themed issue ‘From DNA barcodes to biomes’.

## 1. Introduction

Generating an accurate ‘Tree of Life’ (phylogeny) including every species that exists, and has ever existed, is an impossible challenge. However, systematists work toward this goal, adding parts of the puzzle taxon by taxon. The culture of science includes incentives to work both independently and cooperatively. In contrast with endeavours like the space programme, which cannot be advanced without constant coordination, there is a logical subdivision of labour in systematics, with specialists working, sometimes in isolation, on their own phylogenetically organized taxa, gradually adding consensus and modifying hypotheses, which we hope improve with time as we add both characters and taxa. Our independent work can eventually come together. A sense of urgency has been expressed about the pace of ‘completing’ the Tree of Life. For example, in the USA, the National Science Foundation's ‘Assembling the Tree of Life’ and ‘Dimensions in Biodiversity’ programmes have attempted to accelerate the pace by supporting collaborative efforts with large grants. The challenges are significant. Recently, it was thought that a major bottleneck towards completing the Tree of Life would be in computing speed, but programs such as RAxML [1] and FastTree [2] permit the rapid and efficient analysis of datasets consisting of thousands of both genes and taxa. The ultimate goal under one way of thinking would be to develop the ability to analyse millions of species simultaneously and summarize them on the same comprehensive tree. A more practical alternative approach would be to subdivide the tree into smaller taxonomic groups, as has been standard practice, with each specialist working on their own group. With this approach, subtrees must be grafted together into a larger Tree of Life. A recent study proposed to align phylogenies from different resources to synthesize a comprehensive Tree of Life, i.e. the Open Tree of Life [3]. We believe, however, that it is critical to identify monophyletic groups carefully *a priori*, to avoid unstable phylogenies for subsequent analysis. The strategy of subdividing the task into smaller subclades is a solution that simply requires some criterion for deciding which clades can be independently analysed.

Systematic biology is facing a radical transition from the standard ‘few genes and morphology’, PCR-Sanger-based approach, to transcriptomic and genomic sequencing. With new datasets of unprecedented size (e.g. [4–6]), the backbone of the Tree of Life, at least for insects, has been largely resolved. This is a good time to discuss how we might integrate the work on the terminal branches of the tree, such as the DNA barcoding efforts [7]. A balance must be struck between using millions of nucleotides from transcriptomes—representing only dozens of representative taxa—and using barcode data from, for example, 40 000 individuals.

### (a) Using DNA barcodes to build leaves of the Tree of Life

Using DNA sequences to identify specimens has been a possibility since the 1980s. However, initial efforts were uncoordinated and without standard and agreed-upon protocols (i.e. [8,9]). A truly grand vision of using DNA to identify every species on Earth would require a coordination of efforts, with the selection of one, or a few standardized gene fragments, and a huge database of identified sequences and collections of voucher specimens, to which an unknown specimen could be compared and then identified [7,10]. A coordinated international effort (the International Barcode of Life, or iBOL, http://www.ibol.org/) to create such a database (Barcode of Life Database, BOLD, http://www.boldsystems.org/) has been underway for over 10 years. A 658-nucleotide fragment of the 5′-end of mitochondrial cytochrome oxidase c, subunit 1 (COI) is the most commonly used marker for identifying animal species. This short, standardized DNA fragment is referred to as a ‘barcode’.

The BOLD is a powerful tool for organizing, visualizing and downloading DNA sequences, images and collection records, and we have made extensive use of it here. By February 2016, over 6 million specimen records had been registered in BOLD, representing over 250 000 species and approximately 500 000 BINs. In addition to its intended function for species identification, we present a constructive, integrative approach to discovering, describing and understanding biodiversity, using Trichoptera, or caddisflies, as a model taxon. We are particularly interested in Trichoptera taxonomy and phylogenetics. We recognize the limitations of barcode data for generating phylogenies [11,12] and discuss how these limitations might be mitigated for both inferring phylogeny and for discovering and describing biodiversity.

### (b) Using Trichoptera as a model system

Trichoptera are an order of holometabolous insects, with small, moth-like adults and aquatic larvae that produce silk to construct a diverse array of case and retreat architecture [13,14]. The larvae exploit diverse aquatic microhabitats and are important participants in nutrient dynamics and energy flow in freshwater ecosystems. Because different species are differentially sensitive to pollution, their relative diversity and abundance can be used to assess and monitor water quality [15]. It is the larval stage in Trichoptera that is collected for biological monitoring, but because most taxonomy (i.e. species diagnoses and descriptions) is based on male genitalia, many larval species (and females) are difficult to identify and have not been described. We are fortunate in that the monophyly of Trichoptera is well established and its sister taxon relationship with Lepidoptera (moths and butterflies), comprising the super-order Amphiesmenoptera, is the most strongly supported sister order relationship within insects [16]. Therefore, a phylogeny of Trichoptera can be inferred and subsequently rooted with its sister taxon Lepidoptera alone. Then the Amphiesmenoptera phylogeny can be grafted onto a larger phylogeny, derived from an analysis of Holometabola, using representative taxa, rather than all species. Still, generating the phylogeny of Trichoptera, a moderately diverse insect order with over 14 500 described species, is no small task. The most significant challenge is the availability of specimens representing the order's diversity. Most species (and higher taxa) follow a hollow curve distribution [17], referring to the dominance of few abundant species and the presence of many more rare species, some of which may be known as single specimens, collected only once and never seen again. Since 1995, with the help of collaborators, we have collected molecular data from approximately 480 of the 616 extant Trichoptera genera. This includes sampling a leg from every Trichoptera species (under a sampling threshold of 40 years old or less) in both the National Museum of Natural History (Smithsonian Institution) and the University of Minnesota Insect Collection (UMSP). These institutions have employed Trichoptera specialists for 55 and 30 years, respectively. Other specimens have come to us from H.M., who holds one of the largest Trichoptera collections in the world. If one were to combine all the collecting efforts from our previous Trichoptera phylogenetics projects, it would conservatively be represented by over 100 ‘person years’. Yet over 20% of the genera are missing, many of which will probably remain unobtainable (e.g. *nomina dubia* or rare, monotypic genera). According to the Trichoptera World Checklist [18], as of January 2013 there were 14 548 extant species of Trichoptera, making it the seventh most species-rich of the 30 insect orders. There are probably another 15 000 yet to be described (our minimum best professional estimate). While it may be possible to imagine a ‘Tree of Life’ for mammals or birds, the scale of the endeavour for most of life's representatives, insects and other arthropods as well as bacteria, is far greater. However, even if our efforts represent only one-eigth of the Trichoptera tree, we believe it is worth putting together a phylogeny from the taxa that we have now. The overwhelming majority of species in our dataset are represented with only the barcode data. Many of these species have never been subjected to any kind of formal phylogenetic analysis. Here, we reflect on what we are doing with Trichoptera, toward reaching our phylogenetic goals, and suggest that our approach may be applicable to other taxa.

Earlier work on a single diverse genus, *Chimarra* [12], showed that many nodes from COI alone were congruent with our best estimates of phylogeny from multiple genes and morphology, and those that were ‘unexpected’ were weakly supported and easy to identify. Closely related species clustered together with high bootstrap values at the tips and deeper relationships were also recovered in congruence with morphological and nuclear DNA data. An area of incongruence was also clear between the nodes at the tips and the deeper nodes, with intermediate nodes showing morphologically unexpected or geographically surprising relationships with weak support [12]. Therefore, the phylogenetic results from barcodes were considered mixed. Despite conflict, the barcode data were promising in that a small amount of rRNA data (estimated to be more appropriate for capturing intermediate and deeper nodes [11]) seemed able to dominate the combined data. In other words, although the number of variable characters from the barcodes was far greater than the rRNA, the barcode data did not appear to carry any strong biases, and was able to inform on the tips of the tree without negatively influencing deeper nodes. Even though it is clear that COI is not an optimal gene for deep-level phylogenetics [11] and single genes may not reflect species phylogeny (gene trees may not match species trees, especially at the shallow parts of the tree toward the terminals) owing to independent sorting, introgression or other problems [19,20]), the relevant question is: ‘Are COI-generated phylogenetic hypotheses worth reporting?’ We show that they are.

## 2. Material and methods

### (a) Dataset

The public records from the BOLD systems were searched for Trichoptera in February 2016, using the following criteria: more than 500 bp, not flagged as errors, contaminants or stop codons. Once the FASTA files were downloaded from the BOLD website, we applied a script that identified and merged all identical haplotypes into a single OTU (operational taxonomic unit, which in this context is equivalent to a unique haplotype). Additional scripts were written to reduce the taxon labels provided by BOLD to include only the species name, a three-letter locality abbreviation indicating ‘country’ and numerical codes that indicate the number of individuals that possess that identical haplotype. The seven largest countries (by area) received a second two-letter abbreviation for state or province. If individuals with identical haplotypes were collected in different locations, each location is represented in the taxon name. We uploaded all relevant files to a GitHub repository (https://github.com/pbfrandsen/trichoptera_barcodes), including all scripts and detailed sample information. Specimen IDs are recorded according to their labels on the tree and available as an Excel file on GitHub. Specimen information, along with sequences, electropherograms and primer details for each specimen are available in BOLD at the DOI dx.doi.org/10.5883/DS-TBOL and in GenBank (accession numbers KX291053--KX296688).

### (b) Phylogenetic inference

#### (i) Phylogenetic constraints

Because the COI gene has been shown to be homoplastic for recovering deep-level phylogenetic trees for Trichoptera [11,12], we applied a series of topological constraints to our analysis to concentrate the resolving power of the barcode data toward the tips of the tree. These constraints were generated from a variety of sources, using a variety of criteria. The primary source of constraints came from our most recent phylogeny [21], generated from multiple genes, and over 10 000 nucleotides; nodes from this work that had bootstrap values above 85%, and/or nodes that were supported by two or more independent loci were constrained. Thus, in this case, independent corroboration was deemed more important than bootstrap support, but in reality, these measures are highly correlated [21]. We also used other smaller datasets (cited below), and for these, we set the constraints for nodes supported by posterior probabilities of 100%, because posterior probabilities are often much higher than bootstraps [22]. Additional taxa from Malm and co-workers [23] were added to the whole order constraint tree. Within individual families, we set constraints for Leptoceridae [24], Polycentropodidae [25] and Glossosomatidae [26], and within genera, for *Chimarra* [12]. The constraint tree is available as a Newick file in the GitHub repository.

#### (ii) Phylogenetic reconstruction

Once sequences were downloaded and constraints were established, we analysed them with RAxML [1], under the GTR + GAMMA substitution model. First, we estimated a tree representing all OTUs within Trichoptera. The constraint tree described above was used to guide the tree search via the ‘-g’ option in RAxML, which allows the user to specify a multifurcating constraint tree for a subset of the taxa or all taxa in the alignment. Taxa not represented in the constraint tree were then placed into the scaffold phylogeny induced by the constraint. The taxa not present in the constraint tree are therefore placed by the barcode data, but because the constraint tree is fixed, it is probable that new taxa will attach themselves to a taxon in the constraint tree that is relatively closely related. This strategy works best when the constraint tree is densely populated.

Next, in a separate analysis with different input taxa, we generated trees for smaller, monophyletic groups within Trichoptera (usually families). For each of these groups, we downloaded the barcodes from BOLD, including several outgroups. Then we merged duplicate haplotypes and encoded the taxon labels using the same method and scripts that we used for the order-wide tree. We aligned the sequences in MUSCLE [27] and then partitioned each group into four subsets using a site-specific rate model described by Kjer & Honeycutt [28]. The best-known trees were then estimated in RAxML with 1000 rapid bootstraps (using the -f option) [1]. Thus, there are two separate analyses: one for the all taxon tree and another for subgroups. In the all taxon tree, if there is a contaminant that is labelled, for example, as ‘Xiphocentronidae’, but it is in reality an Ecnomidae, it will be placed with the Ecnomidae in the big tree. By contrast, it will appear as an extremely long branch (and obvious red flag) on the Xiphocentronidae tree, because it is not a xiphocentronid [29].

#### (iii) Tree presentation

Presenting all the trees generated for this paper in print (even only for unique haplotypes), with a readable font size, would require nearly 160 pages. This demonstrates one of the challenges in presenting the ‘Tree of Life’, even for a moderately sized insect order with incomplete taxon sampling. We solved this problem in two ways: first, all trees were uploaded to the GitHub repository in Newick format. Each can easily be downloaded and viewed with a program such as ‘FigTree’ [30] or ‘Dendroscope’ [31]; second, the larger (all taxon) tree is also available on the iTOL website (http://itol.embl.de/tree/16011125417288281456757921).

## 3. Results

### (a) The dataset

**Figure 1.**
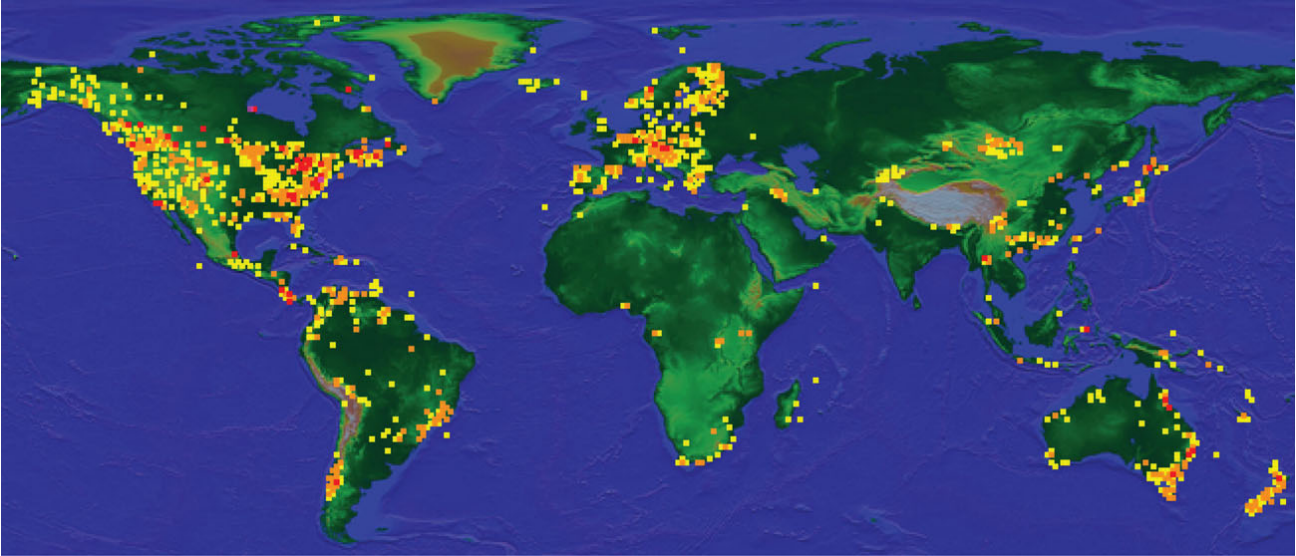
Geographical distribution of public Trichoptera records from the BOLD website (2016). Yellow, 1–9; orange, 10–99; red, 100–999 sample records.

A total of 49 932 records were recovered from BOLD, of which 38 999 barcodes met our criteria. This dataset includes 5569 ‘BINs’, representing 3280 named species (including 33 subspecies) and 1009 interim names (provisional identifications used by BOLD users, a system often applied by taxonomists as working hypotheses to highlight potentially new or unidentified species), from 484 genera and all 49 Trichoptera families. A ‘BIN’ is a DNA-barcode-based registration system for animals, which represents a cluster of haplotypes, aiming to facilitate revisionary taxonomy [32]. Specimens came from 81 countries, with concentrations visible on the map generated from BOLD (figure 1). Viewing the BOLD taxonomy browser in February 2016 for Trichoptera (http://www.boldsystems.org/index.php/Taxbrowser_Taxonpage?taxid=99), we see a concentration of sampling efforts at the Biodiversity Institute of Ontario (BIO, 19 783: 34%), the University of Minnesota Insect Collection (UMSP, 8301: 14%), the National Museum of Natural History, Smithsonian Institution (NMNH, 6,376: 11%) and others (23 733: 41%). However, the number of individuals processed and the number of species added to the database are not tightly correlated because many of the individual samples come from bio-survey efforts, sometimes with hundreds of individuals of the same species. Collections from the UMSP and NMNH were specifically sampled by specialists to increase the taxonomic diversity of the database, as were the samples prepared by K.M.K., from collaborators. Eighty-one countries are listed in the BOLD website, but Canada and the USA account for 59% of the individuals. Australia, Costa Rica, China, Germany, New Zealand and Ecuador, listed in order of numbers, each provided over 1000 specimen records, adding another 21% to the list.

These numbers reflect the significant focus on the Canadian fauna, from the Biodiversity Institute of Ontario in Guelph; the collecting efforts of R.W.H. (UMSP) and O.S.F. (NMNH) in the USA and the Neotropics; X.Z.'s efforts in China; Dave Ruiter's collections in western USA; and significant barcode initiatives in Australia and New Zealand, led by M.S., B.S. and I.D.H. Each of these centres of effort is visible on figure 1, as are the homes of individual collectors, such as J.C.M. at Clemson University (SC, USA), and H.M. in Austria, who has also worked extensively in Thailand. A significant effort was also made in Northern Fennoscandia by J.S., and J.C.M. and S.C. collected extensively in Mongolia. The entire continent of Africa is represented by only 398 samples. Notably missing from the dataset, India has only 28 records. Schmid's extensive available collections from India are over 50 years old and although it may be possible to obtain barcodes from these specimens, it has not been seriously attempted with specialized techniques that would probably be required. Most standard procedures for sampling Schmid's material resulted in failures, and in the time since Schmid's collecting trips, India has imposed severe restrictions on the collection of DNA data by foreigners.

An exciting feature of the BOLD website is that ‘keyhole map language’ (.kml) files can be downloaded, so that collection localities and images for each individual (when available) can be visualized in the ‘Google Earth’ program. A .kml file for this work is available for download from the GitHub repository. All barcodes and associated meta-data as well as haplotype labels presented on the barcode phylogeny are downloadable as an Excel file (Taxon_metadata.xlsx) available from the GitHub repository.

### (b) The Trichoptera barcode phylogeny

**Figure 2.**
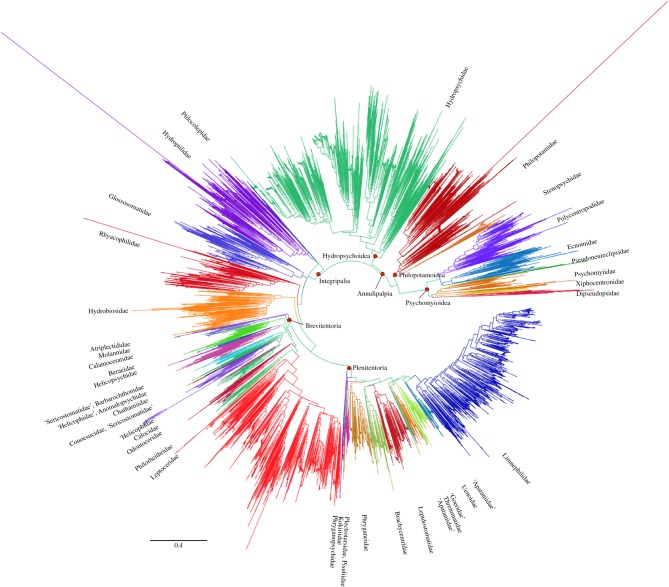
Circular phylogram of constrained barcode phylogeny. Colours represent families of Trichoptera. A vector format is available from the GitHub repository (https://github.com/pbfrandsen/trichoptera_barcodes).

The results from our work on *Chimarra* [12] encouraged us to employ DNA barcodes in phylogenetic reconstruction (but with caution). Our phylogeny for the entire order is shown in figure 2. There are two ways to visualize the details of the tree. First, a Newick file of this tree is available for download from GitHub and can be viewed in phylogenetic tree viewing software. Second (recommended), the tree is visualized on the iTOL website (http://itol.embl.de/tree/16011125417288281456757921). This file will be periodically updated by the Trichoptera Barcode of Life community. The tree shown in figure 2 will be labelled as ‘Trichoptera_barcode_tree_28_Feb_2016’.

Another resource available for download is individual trees for trichopteran families. Compared with the larger tree, errors in these trees are easier to detect. For the larger analysis (figure 2), RAxML was given a tree with individual haplotypes as a backbone, upon which the RAxML program grafted additional haplotypes where they best fit without changing the order of the branches on the constraint tree. This is ideal, because if a specimen is misidentified, or is a contaminant, then it will attach to the tree by what it is, not what it is named in the database. Most of these misplaced taxa are the result of errors in data submission, or errors in identification (most often females or larvae), or unidentified contamination errors, as suggested by long branches in figure 2. These long branches are intentionally retained. It will take years to clean up the entire dataset, as it is continually growing and corrections often involve specimen loans and careful examination of the vouchers. We do not think it wise to throw some out without a clear criterion for doing so. In addition, we contend that these errors need to be visualized before the painstaking process of curating the dataset is completed. For example, we noted that some of the samples from far Eastern Russia have barcodes that are identical across families. While re-examining the Excel sheets we used to submit these data, we found a transposition error that resulted in some taxa being mislabelled. These errors are obvious in the tree file and will be corrected in subsequent online revisions.

### (c) Using barcodes for integrative taxonomy

**Figure 3.**
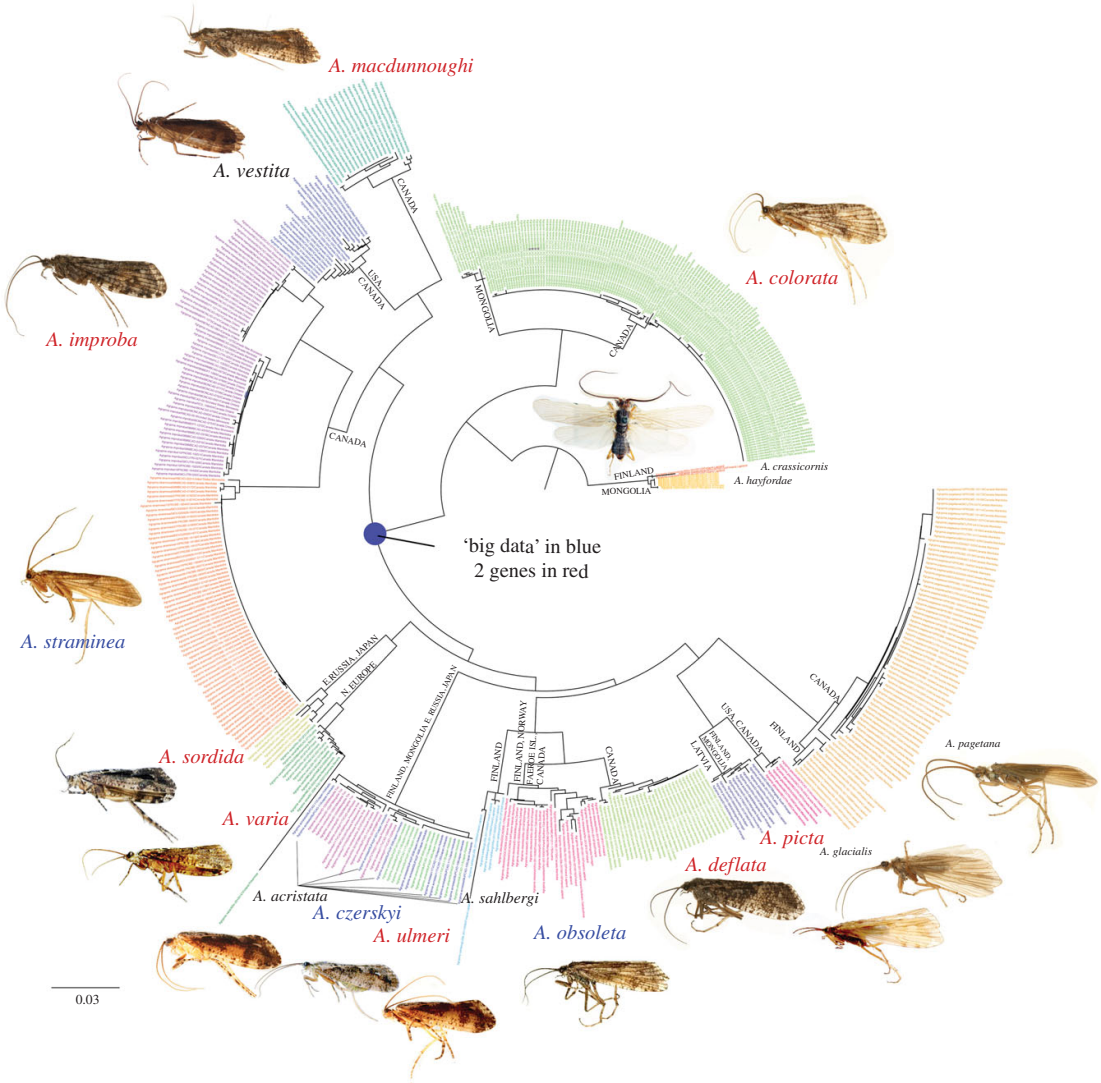
Phylogeny of *Agrypnia*, demonstrating the concept of using barcode data to improve the taxon sampling at the tips of the tree, and using larger datasets as topological backbone constraints. Species names in blue: those represented by hundreds of genes; species in red: those represented by COI barcode and ribosomal DNA; species names in black: those only represented by COI barcode.

The large Trichoptera barcode phylogeny tree provides an invaluable basis for careful scrutiny of species boundaries and relevant hypotheses. For instance, figure 3 provides an example of integrating barcode data into a previous phylogenetic hypothesis. Here, morphology, traditional Sanger-based molecular analysis and transcriptome data placed *Agrypnia* within Phryganeidae [21]. We generated a large dataset of Phyganeidae [33] that included three *Agrypnia* species, *A. straminea, A. obsoleta* and *A. czerskyi*, covering 892 genes. *Banksiola* and *Oligotricha* were selected as outgroup taxa [33]. We also generated 28S sequences for seven additional *Agrypnia* species, all of which had barcode data. Finally, we added the barcode data to the backbone constraints generated by the larger datasets.

When examining the phylogeny (figure 3) at the species-level, we see many species whose haplotype clusters are distinct, and reflect the names they have been given. Most of the species in figure 3 are preceded by long internodes that reflect genetic isolation that match our conceptual ideas about what species are. However, others are a mixture. The barcode haplotypes for *A*. *czerskyi*, *A*. *cristata* and *A*. *ulmeri* are intercalated among one another (figure 3). This does not necessarily mean that they are not real species. Species are defined by taxonomists using integrative datasets, not barcodes alone. However, figure 3 should encourage a taxonomist to reconsider the evidence for this cluster of near identical barcode haplotypes.

## 4. Discussion

In this paper, we presented the largest Trichoptera phylogeny to date, which was built based on data from multiple genes for deep-level nodes and novel barcode data for terminal tips. This large phylogeny (figure 2) contains a great deal of potentially useful information. For example, taxonomists working with any species in the tree can find hypothesized sister species that are probably good outgroups. Both taxonomic and geographical consistency clearly demonstrate that this is a meaningful tree. In our most recent phylogeny [29], the barcode fragment alone recovered Trichoptera, Annulipalpia, Integripalpia, Brevitentoria and Plenitentoria—virtually the entire backbone of deep Trichoptera phylogeny. Similar results were seen across Trichoptera [34] and in *Chimarra* [12]. However, all these studies noted that intermediate nodes were problematic when assessed by congruence to morphology and/or other molecular data. A thorough discussion of ‘why?’ is beyond the scope of this paper, and it is difficult to separate nodes we ‘like’ from those we do not. Still, the tree is likely to contain two sources of inaccuracy. First, like all phylogenetic hypotheses, our constraint tree is likely to contain errors. Constraints were built from much larger, published datasets and can be modified as our future understanding improves. Second, inaccuracies can arise from the limitations of the COI data itself. The barcode is, of course, only a short fragment of a single, rapidly evolving locus. We suspect that the analysis of any short fragment of DNA will be subject to stochastic error. In deep parts of the tree, we suspect that the barcode fragment will be saturated [11] (although it still recovers many deep nodes on its own). However, some of these problems are alleviated by the constraints and have little effect on intermediate nodes. Confounding signal from incomplete lineage sorting (at the shallow nodes) or historical hybridization and introgression (at the deeper nodes) is a possibility. We note that these are not ‘errors’, but rather, a reflection of true biological processes when they are accurately estimated. However, they are in conflict with the species tree. For many groups of caddisflies, however, the barcode data provide the only phylogenetic hypothesis we have. How ‘good’ the tree is, especially at the shallow nodes, can only be discovered with additional data. We find these phylogenetic hypotheses to be useful for circumscribing potential species, for inferring a first hypothesis of relationships among species, for identifying misidentified taxa, and for associating unidentified life-history stages with their described adult stages. We are committed to continually updating our phylogeny. As we add more genes, we can address congruence, decrease stochastic errors and increase node support. As we add more taxa, even with a single gene, other problems owing to long-branch effects, for example, will be reduced. Here, we present our current best hypothesis. We would recommend, given the nature of the data, that nodes with low bootstrap values should be ignored or discarded. Contamination, misidentification and misclassification in public databases, including BOLD, are genuine problems that need to be addressed. Here, we provide two means to detect them. They will appear as long-branch taxa in the family-level trees on GitHub (which were analysed together according to the names assigned to them, which are sometimes wrong). A search through the whole taxon tree (figure 2) for those same haplotypes will offer a hypothesis of what they really are, as you might find members of different suborders appearing to have identical, or nearly identical haplotypes. This is because in the big tree, all taxa are analysed together, and mislabelled taxa will find their proper place in the phylogeny, despite the error in the label. Either case should direct researchers toward the voucher specimens for either correction or reclassification.

There have been attempts to automate tree building, tapping into public databases to produce large summary phylogenies. For example, researchers have explored the possibility of producing trees from GenBank of up to 8000 taxa [35,36]. However, these trees were subject to the errors in the database (as indicated above) and the limitations of the few genes that dominate in GenBank. We find the prospect for non-specialists to produce an automated phylogeny at the push of a button to be exciting, but such a phylogeny is only as good as the care that went into producing the database and in the rigour of the phylogenetic analysis. Our recommendation is a distinct alternative, in that we think specialists should evaluate phylogenies from reliable sources, to update current hypotheses based on constraints, using specified, transparent criteria. These constraints can then be used as a backbone on which to hang other data such as barcodes. Barcodes are ideal for what they were designed to do: distinguish species, and even populations, from one another. However, phylograms with extremely long terminal branches relative to short intermediate and deep internodes are problematic for phylogenies [11], and the BOLD tree option, which uses Neighbour-Joining [37], was not designed to produce phylogenies (although it is excellent for finding the closest match across the datasets, which is what it was designed to do). We decided which constraints were reasonable and justified this based on our expertise in Trichoptera. The advantage of an expertly curated phylogeny available on the World Wide Web is that an up-to-date phylogeny will be available beyond the print version of this paper, and for as long as the authors are able.

### (a) A plan for future phylogenetic work

**Figure 4.**
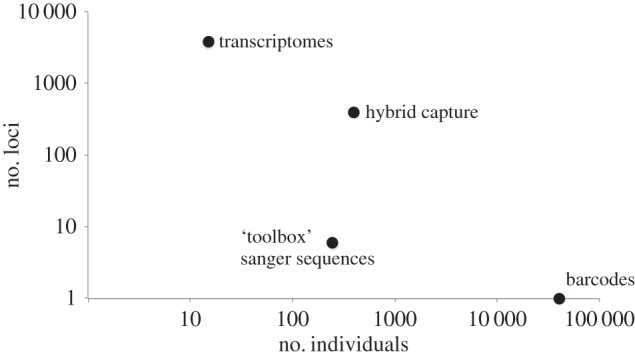
Comparison of dataset sizes in terms of number of taxa and number of loci.

In Trichoptera phylogenetics, there are four sources of molecular data that are quite different in scale, each targeted at different levels of divergence: transcriptomes, targeted enrichment sequences [38,39], PCR-based Sanger nuclear sequences and barcodes. We have generated a data matrix from six of the standard ‘toolbox’ genes, comprising approximately 12 000 combined nucleotides for approximately 250 taxa [11,40,41]. Others have also made significant contributions to the higher-level phylogeny of Trichoptera and their work also contributed to the constraint tree [23–26]. Transcriptome data are being collected through the 1000 Insect Transcriptome Evolution project (1KITE, www.1KITE.org), and these data dwarf the previous dataset, with 3800 nuclear genes for 60 trichopteran taxa (figure 4). However, transcriptome sequencing requires the collection of fresh, specially preserved specimens, which makes the inclusion of rare taxa difficult. To fill these taxonomic gaps, we have been generating data with hybrid capture techniques [38] and have captured 900 loci for specimens representing 250 Trichoptera genera. A strategy for using these large datasets to provide a backbone phylogeny was briefly discussed elsewhere [33]. It should be possible to generate transcriptomes from every family, hybrid capture data from every obtainable genus, rRNA (ideally the 28S, D2) from thousands of species, and barcode data for most species. Our strategy is to use large, multigene datasets to generate a backbone tree, and then use barcode data to fill in the leaves of the tree. It is our intention to continually update this tree as new constraints are discovered through our future analyses of large datasets.

### (b) DNA barcodes and integrative taxonomy

Repeating the process illustrated in figure 3 (for *Agrypnia*) for the other 615 extant genera would be a difficult task, but not impossible to imagine. As datasets grow, particularly from high-throughput sequencing, constraints will improve. Every population does not require a genome to place it in a phylogenetic context. What is missing from our example (figure 3) is taxonomic insight. We believe this would greatly improve the final result and we expect that specialists will contribute summary works on their genera, adding data to the barcodes, as was done with *Chimarra* [12], *Neophylax* [42] and Drusinae [43,44] using two or more genes and morphological expertise.

**Figure 5.**
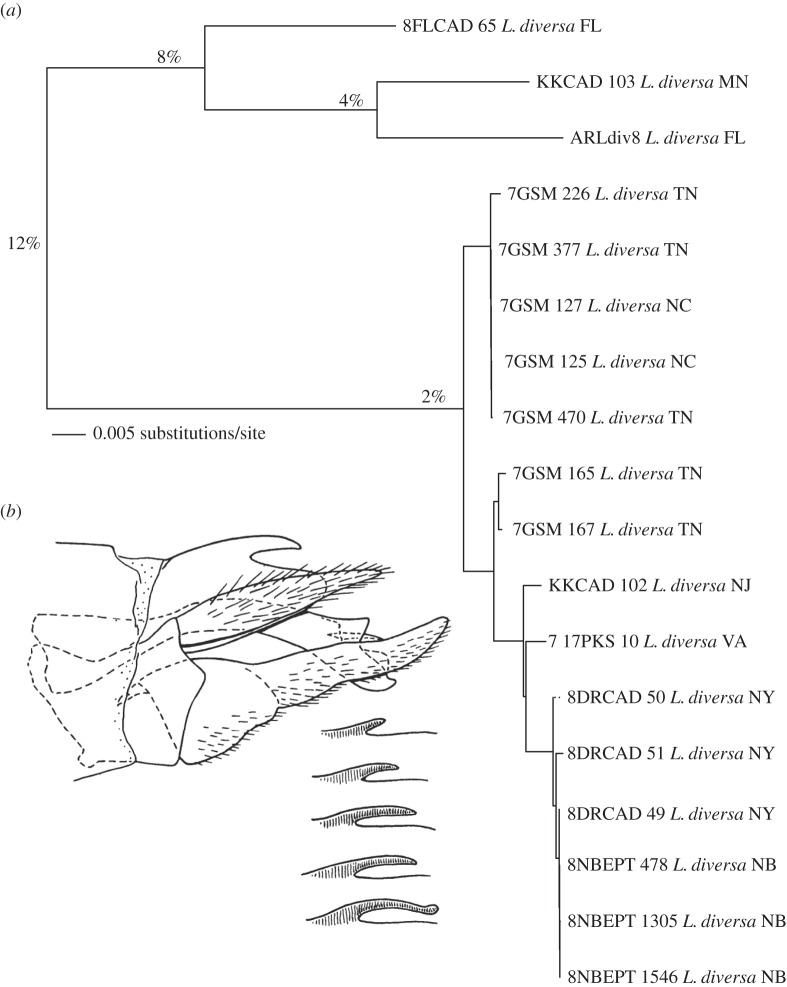
Example of corroborative species illumination. (*a*) Neighbour-joining phylogram from BOLD of *Lype diversa* (Psychomyiidae). Taxa are labelled by an abbreviation of the specimen ID, followed by the species name and then state or province abbreviation. Numerals on the internodes (in per cent) represent mean pairwise distances between the nodes directly to the right. (*b*) Illustration from [45], ‘showing variations of the dorsal horn of the 10th tergite’.

An example of how barcodes can inform descriptive taxonomy is shown in figure 5. There is a hint that morphological variation was recognized from the description, in the species epithet of *Lype diversa* Banks (Psychomyiidae). Ross [45] illustrated variation in the genitalia of this species (figure 5*b*). Still, it has remained a single species since its description in 1914. The variation in branch-lengths in the barcode phylogram points toward hidden biodiversity. Using a 2% threshold, a number that is often correlated with species-level diversity, there are five species. However, biological species in reality are not based on pairwise haplotype divergence. Useful algorithms can be constructed that estimate mean differentiation within circumscribed lineages, and these algorithms are useful for estimating diversity in biological sample assemblages, as well as the probable number of species clusters in aggregate. But on the individual level, species should not be defined on expedient algorithms. We recommend that haplotype clusters (as shown in figures 2, 3 and 5) can be explored by taxonomists in search of species hypotheses. For example, if KKCAD-103 and ARLdiv8 shared a straight stub on the 10th tergite that looked like the top illustration in the inset to figure 5*b*, and 8FLCAD-65's spine looked like the spine at the bottom, while all the rest had curved, but not elongate spines, lacking the knob on the end, then with confirmation of these patterns from museum specimens, and consideration of geographical patterns, a taxonomist could confidently describe three species from figure 5, secure in the application of a species definition that matches our concepts of speciation. However, to revise the *L. diversa* species complex, additional specimens should be examined across the range of the species, the morphological characters should be described and analysed in detail, the type species and any synonyms should be examined to determine which available names apply to which morphotypes, the new species and their variation should be carefully illustrated or photographed, new names should be proposed as needed, and specimens should be vouchered, type specimens designated and all specimens deposited in a publicly accessible museum. These requirements are difficult in today's funding climate and we are dismayed at the continued erosion of support for taxonomy.

The patterns illustrated in figure 5 are not uncommon. The classic paper by Hebert and co-workers [46] is a prime example of cryptic biodiversity discovery. Years of observations on larval diet and morphology of *Astraptes fulgerator* (Lepidoptera, Hesperiidae) did not lead to serious species descriptions (ignoring the precedent of Brower [47], which we would not follow as an example), because the adults were indistinguishable, and in this case, it was difficult to decide if the observed variation was the result of environmental factors. It is the task of taxonomists to decide if the variation they observe is intraspecific or interspecific. In the above given example, Brower proposed to accept formally 3 to 7 of the 10 barcode clusters at species rank [48]. Barcode data provide corroborative evidence, but cannot stand alone without reciprocal illumination from other genes and/or morphology. A similar example was shown by Harvey and co-workers [49], who found that distinct haplotype clusters precisely matched fixed variation in larval head morphology of the *Diplectrona modesta* species complex, a widely distributed caddisfly across eastern North America (see their fig. 2).

We have witnessed an alarming decline in the support for museums and organismal taxonomy. The Trichoptera barcode database owes much of its utility to the tireless efforts of specialist taxonomists collecting and identifying specimens. Without these identifications, the database would show only a large collection of DNA haplotypes without associated species names. Species names have been a baseline for biological work for more than 250 years, and should not be abandoned for BINs or MOTUs (Molecular Operational Taxonomic Units). Earlier work on Trichoptera has shown that the number of haplotype clusters in an environmental sample is tightly correlated with the number of species [50], so that BINs in aggregate can provide information that can be decoupled from taxonomy. For some applications, such as biodiversity inventories, this correlation would translate into fast, efficient and accurate biodiversity assessments and community ecology studies. However, abandoning the process of species description altogether for the expediency of single-gene based BINs would be a mistake. Barcode data can, however, assist in corroborating morphological species boundaries, as shown in figure 5, and demonstrated by e.g. the work of Flint & Kjer [42] and Previšić and co-workers [43]. As the barcode database grows at its own rapid pace, it will be increasingly valuable in distinguishing between intraspecific and interspecific variations [51]. The two systems (generation of barcode libraries and the description of species) can advance independently, but should not be entirely decoupled. Haplotype clusters can be algorithmically defined, and should be called ‘BINs’ (where obtained from BOLD's BIN System) or ‘MOTUs’ from other molecular analysis algorithms. Congruent genetic clusters from multiple genes or whole genomes are probably distinct species, but we should still refrain from calling them species until they have been formally described by taxonomists. An example of the merging of DNA taxonomy with biologically sound species concepts was presented for Chinese Hydropsychidae; these authors made recommendations for associating larvae with adults that coupled congruence across multiple genes with morphological hypotheses of species [20]. These kinds of associations can be used to describe the morphology of the larvae to aid the use of these species in water quality assessment [50,52,53]. Similarly, barcode evidence, together with morphology, has been employed in differentiating cryptic diversity and defining new species of South American and European caddisflies [43,54,55]. Thus, we find that barcodes provide a valuable tool in answering a wide variety of ecological and taxonomic questions. However, without support for taxonomy, where expertise is rapidly declining, we condemn ourselves to see ‘biodiversity’ as a collection of plastic tubes, named with alpha-numeric codes that are divorced from biological context.
